# Basic Properties of Calcined Underground Ant Nest Materials and Its Influence on the Compressive Strength of Concrete

**DOI:** 10.3390/ma12071191

**Published:** 2019-04-11

**Authors:** Wei Zhou, Peng Zhu, Wenjun Qu

**Affiliations:** Department of Structural Engineering, Tongji University, Shanghai 200092, China; wei.zhou@hotmail.com (W.Z.); pzhu@tongji.edu.cn (P.Z.)

**Keywords:** underground ant nest, calcination, calcined ant nest clay powder (CANCP), pozzolanic activity, strength contribution rate of pozzolanic, compressive strength

## Abstract

Underground ant nests are typically made of soil and rubble mixed with dead plant bodies, ant secretions, and other organic substances. The clay content is high, and the natural clay materials show pozzolanic activity after calcination. In this study, the underground ant nest materials of Iridomyrmex anceps, which is a common ant in the Shanghai area, are calcined and ground, and the material properties of calcined ant nest clay powder (CANCP) are characterized from six aspects: chemical composition, particle morphology, specific gravity, specific surface area, particle size distribution and pozzolanic activity index. The pozzolanic activity of CANCP is evaluated by the strength contribution rate of pozzolanic activity, revealing that CANCP is beneficial to the strength of the mortar system from an early age. The influence of CANCP on the compressive strength of concrete is analyzed using three aspects, namely, content, curing age and calcination temperature, and it is found that the three aspects of CANCP have a strong influence on the compressive strength of concrete.

## 1. Introduction

Ants are excellent architects in the animal world; their nests contain unique structures that feature excellent heat preservation, moisture retention and ventilation function. However, ants have strict demands on the building materials of nests, such as the soil particle size, weight, luster and color. Underground ant nests are typically made of soil and rubble mixed with dead plant bodies, ant secretions and other organic substances, and the content of clay is high. The clay of underground ant nests is composed of earth particles cemented together to form a hard brick-like material. Natural clays typically contain a mixture of clay minerals associated with non-clay minerals. Most natural pozzolans develop their activity from clay minerals such as kaolinite, montmorillonite, and illite. Each type of clay has an optimum calcination temperature range that causes the breakdown of the crystalline structure of the clay and the formation of amorphous silica and alumina [1]. Some pozzolanic active substances are suitable for partial replacement of cement and are natural products, such as pozzolan [2,3], zeolite, and solid waste, industrial by-products, such as slag, fly ash, silica fume, rice husk ash, waste glass [4], broken ceramics [5] and clay brick [6], or low energy products, such as calcined clay [7,8,9] and calcined termite clay [1].

Ants change the physical characteristics of the soil through nesting activities [10], which can result in nutrient redistribution in the soil profile [11,12], increased soil porosity, improved soil water conductivity and permeability, increased soil water retention capacity [13], altered soil bulk density [14], improved electrical conductivity, and increased soil nutrient content and potential for higher crop yield [15]. Underground ant nests also influence the chemical properties of soil; such nests can increase the concentration of organic matter, the N, P and K content [16], and the concentration of K^+^, Na^+^, Ca^2+^, and Mg^2+^ ions in anthills [17] and alter the anthill’s pH [18]. Through decomposition of plant residues, ant activity can promote the release of N, decomposition of organic matter, and mineral and nutrient cycling in plant residues [19]. Such activity can also increase soil organic matter, particularly stable humic acid, which is conducive to the stability of the soil structure and soil fertility [20]. Ant activity can also stimulate CO_2_ production [21] and soil organic N mineralization and increase denitrification in soil [22]. Through nitrogenase activity, ants can contribute N to their habitat [23]. Thus, relative to surrounding ordinary clay, the physical properties and chemical properties of underground ant nest materials are strongly altered. In the present work, we study the basic properties of underground ant nest materials after calcination, and we explore and summarize the material properties of calcined ant nest clay powder (CANCP) using six aspects: the chemical composition, particle morphology analysis, specific gravity, specific surface area, particle size distribution and pozzolanic activity index. The paper discusses whether underground ant nest materials after calcination can partially replace the pozzolanic active substance in cement and the associated influence on the compressive strength of concrete.

## 2. Properties of Calcined Underground Ant Nest Materials

### 2.1. Collection and Calcination of an Underground Ant Nest 

Iridomyrmex anceps belongs to the Iridomyrmex category and is distributed across China. Ant nests are underground, and anthills consists of soil particles, the Pinus massoniana male flower, grass, and frass. Anthills are packed into a grave shape, spherical shape, or irregular shape. In this study, the underground nests of Iridomyrmex anceps were sampled from three locations. 

The optimum calcination temperature and time of clay minerals depends on the types of clay minerals, and clay can exhibit potential pozzolanic properties when treated under appropriate calcination temperatures and times. Clay has the greatest activity when it is calcined at 600–800 °C [24]. Thus, 600 °C and 800 °C were used as the control temperatures for calcination tests. Clay calcined for approximately 1–3 h at controlled temperature has the highest activity [25]; thus, 2 h was used as the calcination control time. The underground ant nest material was sampled in batches of 50 kg, and impurities such as plant dead bodies were removed before preliminary grinding. Samples were placed in a box-type resistance furnace, and four groups of temperature sensors were embedded in the upper, middle and lower parts of the ant nest soil to record temperature changes. The ant nest soil was dark-brown and granular after initial treatment. After calcination at different temperatures, the ant nest soil color changed to brick red due to the conversion of FeO to Fe_2_O_3_ at high temperature under aerobic conditions. The ant nest soil quality at 600 °C and 800 °C after calcination led to masses of 39.25 kg and 38.74 kg and a loss on ignition of 21.5% and 22.5%, respectively. 

### 2.2. Material Properties of CANCP

An SM-500 ball crusher was used for milling of the ant nest soil for 60 min after calcination. The chemical composition of CANCP was analyzed using an X-ray fluorescence spectrometer. The total content of SiO_2_, Al_2_O_3_ and Fe_2_O_3_ in 800 °C CANCP and 600 °C CANCP reached 88.76% and 83.37%, respectively, both greater than the 70% specified in the ASTM standard for concrete mineral admixtures [26]. The results are shown in Table 1. The active component in 800 °C CANCP is greater than that of 600 °C CANCP; thus, 800°C CANCP was used as the reference to test the material properties of CANCP. Many tests showed that the active component of ordinary calcined clay was 80–85%, while the active component in 800 °C CANCP was close to 90%, superior to that of ordinary calcined clay [27,28,29,30].

**Figure 1:**
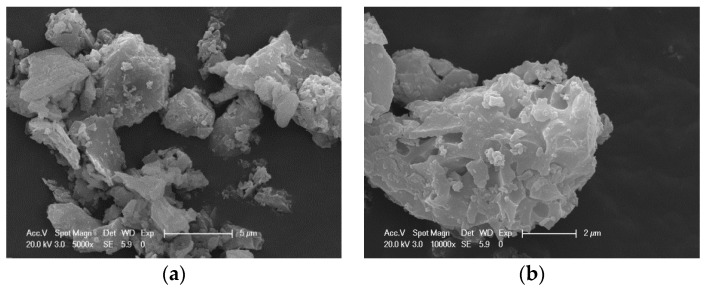
(**a**) 5000× and (**b**) 10,000× CANCP SEM micrographs (calcined ant nest clay powder, CANCP).

After two tests, the margin of 45 μm square hole sieves was measured as 16.25% and 16.58%, close to the 12% requirement of first-grade fly ash [31]. The specific gravity of CANCP is 2.66, and the specific surface area is 491 m^2^/kg. Figure 1 shows SEM micrographs of CANCP at different magnifications. From the particle morphology, CANCP is clearly different from the regular spherical particles of fly ash; most of its particles are angular, and the shape of the structure is not strongly regular, but the particle size distribution is even. As shown in the high-magnification electron micrographs, these particles have cleavage planes, and some fine particles are attached to the surface. This finding indicates that the interior contains some mineral elements that are prone to cleavage and will affect the fluidity of concrete. Through water requirement ratio tests, the water requirement ratio for CANCP is 110% and slightly larger than that of cement. This characteristic has little effect on the flowability of the mixture, and the flowability is slightly worse.

**Figure 2:**
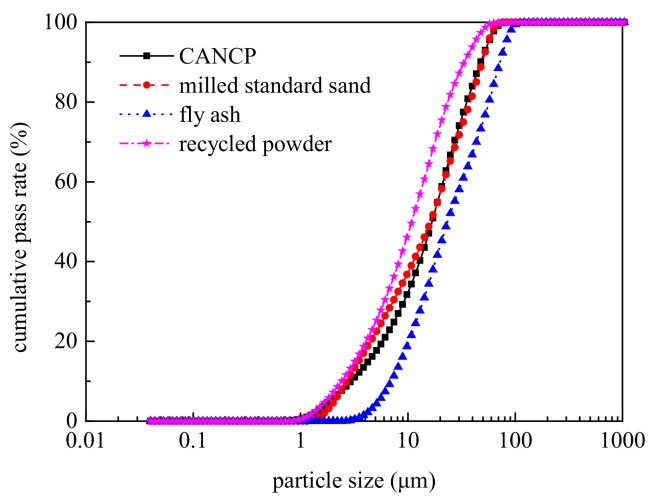
Particle size distribution curves of various materials.

The particle size distribution of CANCP was determined using a Winner laser particle size analyzer. The results are shown in Figure 2. The average particle size of CANCP was 23.35 μm, and most particles were smaller than 50 μm. The particles with sizes 1–10 μm composed a large proportion, and the gradation exhibited continuity. A smaller particle size is better for stimulating the activation of materials; thus, the presence of 1–10 μm particles in a large proportion is favorable for maximizing activity.

### 2.3. Pozzolanic Activity Index of CANCP 

The pozzolanic activity index of CANCP of 800 °C calcination was evaluated using two methods: the lime absorption method (direct) and the strength index method (indirect).

#### 2.3.1. Lime Absorption Method

**Figure 3:**
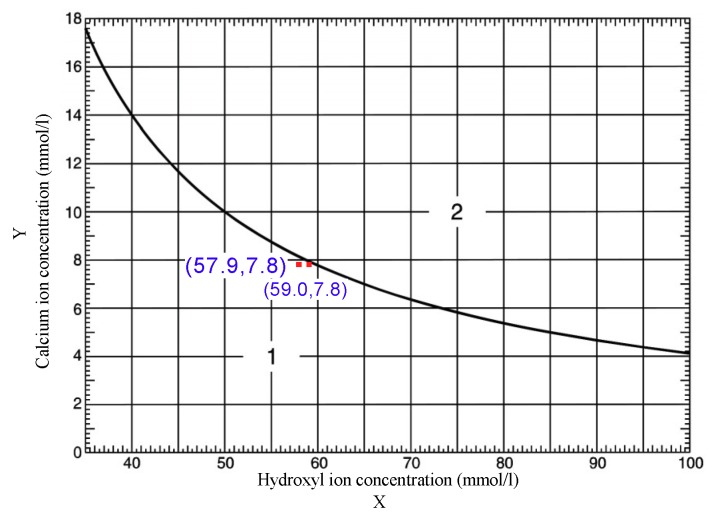
Evaluation of pozzolanic activity graph.

The lime absorption method involves mixing a pozzolanic material with a Portland cement slurry or a saturated lime solution, and the activity of pozzolanic material is quantitatively evaluated by measuring the concentrations of residual Ca^2+^ and OH^−^ in solutions of different ages. Lower concentrations of Ca^2+^ and OH^−^ indicate higher pozzolanic activity [32]. The activity of pozzolanic materials such as fly ash [33], metakaolin [34], and clay brick powder [35] has previously been evaluated using the lime absorption method. Two CANCP samples were taken at random, according to GB/T 18736-2002 [36], and the Portland cement and CANCP were mixed at a 7:3 mass ratio. Two CANCP sample test results were marked on the pozzolanic activity graph, both falling below the curve in the graph (solubility curves of Ca(OH)_2_ at 40 °C). Thus, CANCP material has pozzolanic activity; see Figure 3.

#### 2.3.2. Strength Index Method

The strength index method compares the 28 days compressive strength of the contrast mortar of a certain mineral admixture with an equal mass of cement to that of a reference mortar, and the ratio of these two compressive strengths is defined as the activity index of the composite mineral admixture. Among all mineral admixtures, fly ash is the most commonly used and systematically characterized, and recycled powder can be used to grind waste sintered clay brick to a certain fineness, similar to CANCP; fly ash can also be treated through clay calcination and grinding processes. A comparison of the two types of materials would be meaningful; thus, according to GB/T 18736-2002 [36], CANCP mortar (CAP) and five groups of contrast mortar was designed: reference mortar CT, reference mortar SS, milled standard sand mortar GSS, fly ash mortar FA and recycled powder mortar RP. 

##### Materials

PO 42.5 ordinary Portland cement conforming to GB175-2007 [37] was used; the chemical composition and physical and mechanical properties are listed in Table 1 and Table 2, respectively. The fly ash used in this test was second-grade fly ash produced by the first power plant of Shanghai. The quality of the fly ash was in accordance with GB/T1596-2017 [31], and the chemical composition and physical and mechanical properties are listed in Table 1 and Table 3, respectively.

Recycled powder in this study was prepared by smashing, grinding, drying and grading abandoned clay bricks and cement solids. The chemical composition and physical and mechanical properties are listed in Table 1 and Table 4, respectively. Its density is 2.63 g/cm^3^, and the average particle size is 16.14 μm.

The standard sand was ISO679 standard sand. Milled standard sand was obtained by ball milling of the ISO standard sand for 2 h with a SM-500 ball mill, and the sieving test was performed using a FSY-150E cement fineness negative-pressure sieve analyzer. The margin of a 45 μm square hole sieve was 17.55%, the fineness control requirements are close to that of CANCP, and the average size of the ground standard sand was 23.29 μm. The particle size distributions of CANCP, fly ash, recycled powder, and milled standard sand are plotted in Figure 2. The particle size distribution of CANCP, fly ash, recycled powder, and milled standard sand show few differences. The size distribution curve of CANCP is close to that of milled standard sand, and the curves even overlap in some regions; thus, the fineness of the two is relatively close, and both approach the fineness control requirements for first-grade fly ash.

##### Specimen Preparation and Mixing Design

Specimens for strength index tests were prepared following GB/T17671-1999 [38]. The cementitious materials were mixed for 1 min in the mixer. Then, 70% water was added and mixed for 3 min. The sand was added and mixed for 1 min. The remaining 30% water was added and mixed for 5 min. The mixture was poured into molds measuring 40 mm × 40 mm × 160 mm and consolidated using a vibration table. The specimens were maintained in the standard curing case (temperature 20 ± 1 °C and moisture above 90%) for 24 h. After demolding, the specimens were kept in the curing room (temperature 20 ± 2 °C and moisture above 50%) in preparation for further tests.

The mass mix ratio of reference mortar CT was one part cement, three parts standard sand and half-part water. The reference mortar SS used standard sand instead of 30% quality cement, and the milled standard sand mortar GSS used milled standard sand instead of 30% quality cement. The replacement amount of other pozzolanic ash materials was 30%. The mix design was modified as shown in Table 5.

##### Test Methods 

The CANCP mortar and contrast mortar of each group were cured for 3 days, 7 days, 28 days and 90 days to test their flexural strength and compressive strength following GB/T17671-1999 [38]. Flexural specimens were tested at a loading rate of 50 N/s. The specimens were loaded from their mid-span, and the clear distance between simple supports was 100 mm. The two broken pieces remaining after the flexural test were subjected to compressive strength tests at a loading rate of 2500 N/s. Three specimens were tested for flexural strength. The average of six specimens was used to evaluate the compressive strength of the mixtures.

##### Results and Discussion

**Figure 4:**
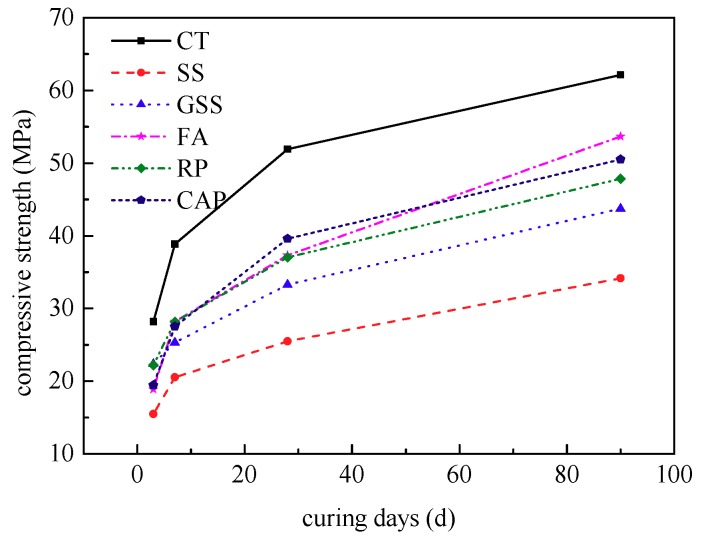
Relationship between the compressive strength of CANCP mortar, reference mortar and contrast mortar.

The test results are shown in Table 6. The relationship between the compressive strengths of the CANCP mortar and the reference mortar, and CANCP mortar and the contrast mortar are shown in Figure 4.

The reference mortar SS uses standard sand instead of cement of the same quality because the standard sand has no pozzolanic activity and because the filling action is weak; thus, the amount of cement decreases. This condition directly results in decreased compressive strength in the mortar, and thus the compressive strength is lower than that of the other mortar after 3 days. The mortar GSS uses milled standard sand instead of cement of the same quality. Milled standard sand also has no pozzolanic activity, but its compressive strength is higher than that of the reference mortar (SS); thus, the micro-filling effect of milled standard sand plays an active role. The compressive strength of CANCP mortar begins to exceed mortar GSS at 7 days, as shown in Figure 2. The fineness of the milled standard sand is essentially the same as that of CANCP, and the filling effect of cement mortar system is also essentially the same. Therefore, CANCP not only provides filling action but also has pozzolanic activity and participates in the hydration reaction of cement. Figure 4 also indicates that the early compressive strength of CANCP mortar is slightly lower than that of mortar FA and mortar RP; the compressive strength at 28 days is higher than that of mortar FA and mortar RP; and the compressive strength at 90 days is higher than that of mortar RP and less than that of mortar FA. 

The activity index of CANCP, fly ash and recycled powder was 76.3, 71.9 and 71.3, respectively, and the activity index of CANCP was larger than that of fly ash and recycled powder and greater than the value of 65 specified in the GB/T2847-2005 standard [39]. These results show that CANCP has pozzolanic activity and meets the requirements for an active admixture.

The pozzolanic activity index obtained from the compressive strength ratio reflects only whether the admixture has pozzolanic activity; the index does not reflect the extent of pozzolanic activity of the admixture. Pu Xincheng [40] introduced the concept of a specific strength of concrete and cement, i.e., the contribution of a unit amount of cement to the strength of concrete, and analyzed the pozzolanic effect of admixtures in concrete. The principle is as follows:

(1) The concrete compressive strength ratio of the mineral admixture *R_ra_*, i.e., the strength contribution of unit cement to concrete, is defined as follows:

(1) $$R_{ra}=\frac{R_{a}}{q}$$

In this expression, *R_ra_* is the specific strength of concrete containing an admixture (MPa), *R_a_* is the absolute value of the strength of concrete containing an admixture (MPa), and *q* is the percentage of cement content in the concrete cementitious material.

(2) The specific strength of reference concrete without mineral admixture *R_rr_* is defined as follows:

(2) $$R_{rr}=\frac{R_{r}}{100}$$

In the expression: *R_rr_* is the specific strength of concrete (MPa); *R_a_* is the absolute value of the strength of concrete.

(3) The difference between *R_rr_* and *R_ra_* is the specific strength of the pozzolanic effect contribution:

(3) $$R_{rp}=R_{ra}-R_{rr}$$

*R_rp_* reflects the contribution of the pozzolanic effect of mineral admixtures to the strength of the entire system. A negative value shows that mineral admixtures reduce the strength.

(4) The strength contribution rate of cement hydration reaction *P_h_* is defined as follows:

(4) $$p_{h}=\frac{R_{rr}}{R_{ra}}\times 100\%$$

The strength contribution rate of the pozzolanic effect *P_p_* is defined as follows:

(5) $$P_{p}=\frac{R_{rp}}{R_{ra}}\times 100\%=\frac{R_{ra}-R_{rr}}{R_{ra}}\times 100\%$$

The pozzolanic activity of CANCP, standard sand, milled standard sand, fly ash, and recycled powder was evaluated using this method. The results are shown in Table 7.

The strength contribution rate of the pozzolanic effect of mortar SS was negative for all ages, and the standard sand had no pozzolanic activity. Thus, standard sand instead of equal-quality cement reduces the strength of cement mortar. The 3 days strength contribution rate of the pozzolanic effect of mortar GSS is 11.73 because it has no pozzolanic activity. For intuitive comparison, the pozzolanic effect strength contribution rate is used to describe its strength in cement paste, and the strength of the pozzolanic effect at this time is contributed by the filling effect of the milled standard sand. The strength contribution rate of the pozzolanic effect of mortar GSS is zero or negative with increasing age, indicating that when milled standard sand is replaced by equal-quality cement, the strength of the cement mortar can decrease, and the contribution of the filling effect of milled standard sand to the strength of the cement mortar appears primarily at early ages. 

The strength contribution rate of the pozzolanic effect of mortar FA increases with age, and the 3 days strength contribution rate of the pozzolanic effect of mortar FA is negative. This result shows that fly ash has an adverse effect on the early strength of the complete mortar system. The strength contribution rate of the pozzolanic effect is positive starting from the age of 7 days, and the fly ash begins to have a beneficial effect on the strength of the entire mortar system; the 90 days strength contribution rate of the pozzolanic effect of mortar FA is 18.96. The strength contribution rate of the pozzolanic effect of the recycled powder RP is positive at all ages, and the 3 days strength contribution rate of pozzolanic effect is as high as 11.27. Thus, the beneficial influence of recycled powder on the entire mortar system is reflected primarily in the early strength, and the 90 days strength contribution rate of pozzolanic effect is 11.93, with a long-term pozzolanic effect that is less than that of fly ash. The strength contribution rate of the pozzolanic effect of mortar CAP increases with age and is positive. CANCP begins to show a favorable effect on the strength of the mortar system from an early age, and the 90 days strength contribution rate of the pozzolanic effect of mortar CAP is 13.91. The long-term pozzolanic effect of CANCP is larger than that of recycled powder but smaller than that of fly ash.

## 3. Compressive Strength Test 

### 3.1. Material 

PO 42.5 ordinary Portland cement conforming to GB175-2007 [37] was used; the chemical composition and physical and mechanical properties are listed in Table 1 and Table 2, respectively. The fine aggregate consisted of natural river sand with a fineness modulus of 2.5, and the coarse aggregate consisted of 5 mm–25 mm continuous graded natural stones; its basic material properties are shown in Table 8 and Table 9. The DY-106 polycarboxylate high-performance water-reducing agent was selected, and the performance parameters are shown in Table 10.

### 3.2. Specimen Preparation and Mix Design

A cubic specimen measuring 100 mm × 100 mm × 100 mm was used to study the compressive strength of concrete following GB/T 50081-2002 [41]. The mold was wiped clean, and a layer of release agent was painted on its inner surface before preparing the specimen. A vibrating table was used with a vibration frequency of (50 ± 3) Hz and a no-load amplitude of (0.5 ± 0.1) mm. The molded concrete block was covered with an impervious film to prevent moisture from volatilization and placed in the indoor environment (20 ± 5 °C) for 24 h. The template was removed, and the test block was numbered. 

In this experiment, CT was the reference concrete, i.e., ordinary concrete, and a standard mix proportion of design strength of C50 was selected. The calculated mix proportions for the ordinary concrete CT were (cement: water: sand: stone = 486:170:741:1023). For the other six sets of concrete, the total amount of cementitious material was 486 kg/m^3^, the ratio of water to binder was 0.35, and the sand content was 42%. The CANCP with 800 °C calcination was used to replace cement in proportions of 5%, 10%, 15%, 20% and 30%, and the CANCP with 600 °C calcination was used to replace cement in proportions of 10% to study the effect on concrete properties. By adjusting the dosage of water-reducing agent, concrete slump was controlled near 200 mm. The mix proportion and concrete slump are shown in Table 11. 

### 3.3. Test Methods

The compressive strength of concrete was tested following GB/T 50081-2002 [41]. The tests were performed immediately after curing for 3 days, 7 days, 14 days, 28 days and 90 days in the standard curing room. The test used a DY-3000DX electro-hydraulic servo microcomputer controlled pressure testing machine, and the loading rate was 0.5 MPa/s. Six concrete blocks were used for each compression test, and the compressive strength was averaged.

### 3.4. Results and Discussion

#### 3.4.1. Influence of the Content of CANCP

**Figure 5:**
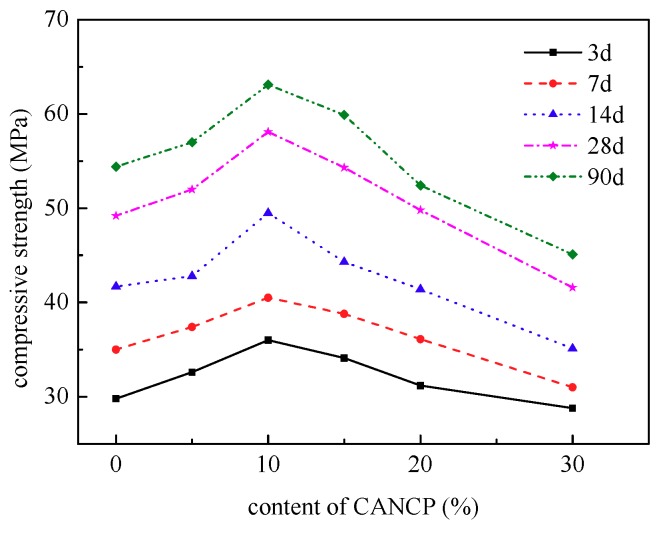
Relationship between the compressive strength of concrete and CANCP content.

For a more intuitive observation and analysis of test results, the ratio of the compressive strength of concrete with varying CANCP content to the compressive strength of reference concrete was used as the relative compressive strength of CANCP concrete. The relationship between the compressive strength of concrete and the CANCP content is shown in Figure 5. 

The content of CANCP has a strong influence on the compressive strength of concrete. With increasing CANCP content, the cubic compressive strength of concrete first increases and then decreases. Each curve has a peak value, and the compressive strength of the concrete containing 10% CANCP is the largest. The compressive strength for curing times of 3 days, 7 days, 14 days, and 28 days increases by 20.8%, 15.5%, 18.9%, and 18%, respectively, and the early strength increases quickly. When the CANCP content is 20%, the compressive strength of concrete is essentially identical to that of ordinary concrete CT without CANCP. When the CANCP content is more than 20%, the compressive strength of concrete begins to decrease, and the compressive strength for 3 days, 7 days, 14 days, and 28 days curing is reduced by 3.2%, 1.6%, 15.8%, and 15.4%, respectively. The early strength decreases less. Adding CANCP to concrete can improve the strength of concrete, but the content should be less than 20%. The optimum content in this experiment is 10% of the cementitious materials.

#### 3.4.2. Influence of Curing Age 

**Figure 6:**
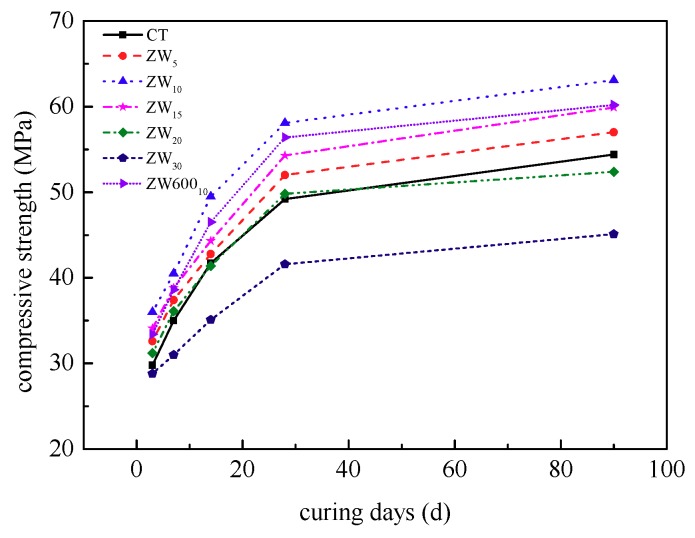
Development curves of compressive strength for concrete with varying CANCP content with age.

The development curves for the compressive strength of concrete containing varying CANCP content with age are shown in Figure 6.

The compressive strength of concrete containing various CANCP content increases with age. The development of early strength is faster, and the 3 days compressive strength reaches the 28 days strength of 60%, the 14 days compressive strength reaches the 28 days strength of 85%, and the compressive strength between 14 days and 28 days of curing is smaller; the 90 days compressive strength is more than 1.05 times the strength at 28 days. To characterize the changes in the compressive strength of CANCP concrete with age, the relative compressive strength of CANCP concrete at different ages was determined by the ratio of the strength at each age to the strength at 28 days, as shown in Table 12.

**Figure 7:**
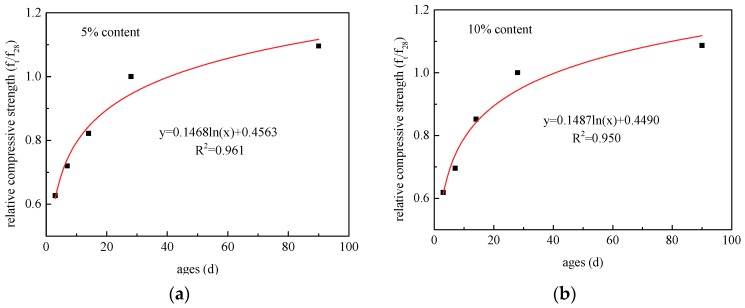

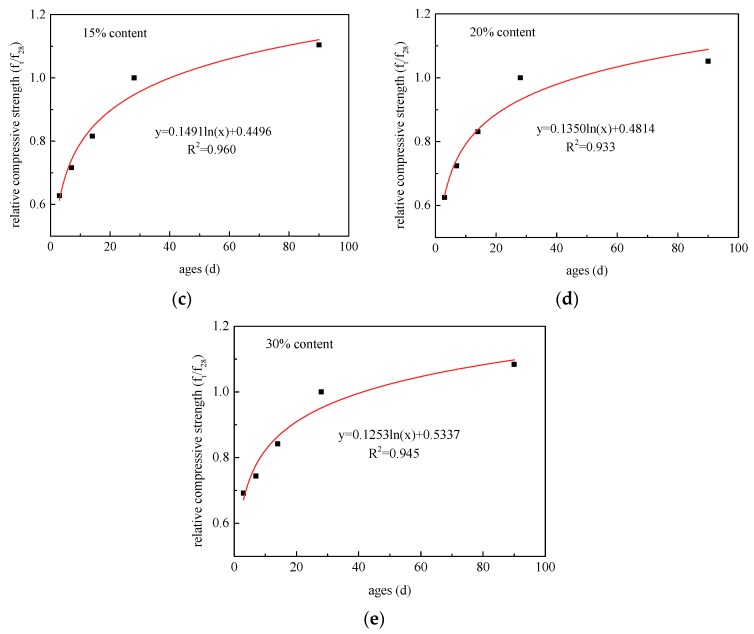
Regression analysis of relative compressive strength and age of concrete with CANCP content of (**a**) 5% (**b**) 10% (**c**) 15% (**d**) 20% and (**e**) 30%.

The ratio of the compressive strength of CANCP concrete at a certain age to the 28 days compressive strength (f_cu_(t)/f_cu_(28)) was defined as the age coefficient of concrete (Y). Using the mathematical model Y = A ln (T) + B, where A and B are constants, regression analysis was performed on the relative compressive strength and age of concrete with CANCP content of 5%, 10%, 15%, 20%, and 30%, as shown in Figure 7.

As shown in Figure 7, the correlation index *R^2^* is greater than 0.93, and the formula obtained by regression is highly correlated. The A and B values for the 5%, 10% and 15% content are relatively close, and the correlation index *R*^2^ is approximately 0.96. The increase in strength with age is essentially consistent when the CANCP content is not more than 15%. 

#### 3.4.3. Influence of Calcination Temperature 

The optimum content of CANCP with 800 °C calcination in this experiment is 10% of the cementitious materials, and therefore, a comparative test of concrete containing 10% CANCP with 600 °C calcination was performed. As shown in Figure 6, the compressive strength of the concrete containing 10% CANCP with 600 °C calcination is higher than that of the reference concrete CT; all ages increased by more than 10%, and the strength at 28 days increased by 14.6%. The strength of all ages decreased by approximately 5% relative to the compressive strength of the concrete containing 10% CANCP with 800 °C calcination.

The CANCP with 600 °C calcination was ground in the same conditions; thus, its fineness was essentially identical to the CANCP with 800 °C calcination and had the same filling effect. Therefore, the test proved that the active component and activity index of CANCP with 800 °C calcination is higher than that with 600 °C calcination. The nest soil is similar to kaolin clay from the analysis of the soil chemical composition, and the differential thermal curve of general kaolin clay is a smooth curve between 700 °C and 800 °C. This finding shows that the thermal effect is low in this temperature range and that the activity is highest at this time [42].

#### 3.4.4. Analysis of Mechanism of Action of CANCP in Concrete

**Figure 8:**
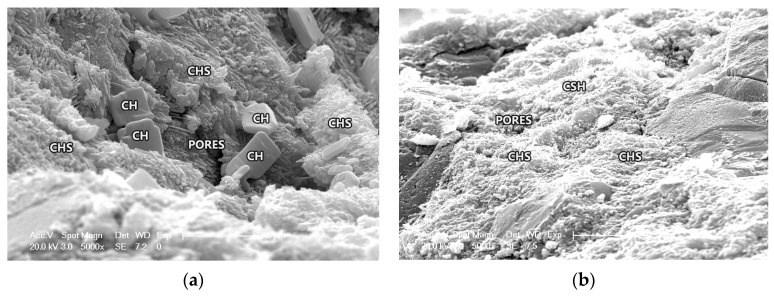
(**a**) 5000× ordinary concrete (28 days curing) and (**b**) 5000× 10% CANCP concrete (28 days curing) SEM micrographs.

The action mechanism of CANCP in concrete can be explained by the three microstructural phases of concrete, which are hydrated cement paste, aggregate, and the interfacial transition zone between cement paste and aggregate. The influence of the interfacial transition zone on the mechanical properties of concrete is much greater than that of other factors [43]. Interfacial adhesion is the key to determining the strength of concrete, and this adhesion depends on the hydration products of cement. The most influential product is C-S-H gel, followed by Ca(OH)_2_. C-S-H gel has a small particle size, large specific surface area, high surface energy and high interfacial bond strength. C-S-H gel has a decisive influence on the concrete strength. Ca(OH)_2_ crystallizes as plates with a large size, small specific surface area, low surface energy, weak interfacial bonding, and a simple orientation arrangement on the surface of the aggregate. Ca(OH)_2_ acts as an isolation effect between the C-S-H gel and the aggregate and plays a negative role in interfacial adhesion. To increase the strength of concrete, the amount of C-S-H gel should be increased, and the amount of Ca(OH)_2_ should be reduced [43]. Figure 8 shows SEM micrographs of ordinary concrete CT and 10% CANCP concrete at 28 days curing under different magnifications.

CANCP has strong pozzolanic activity. The amorphous Al_2_O_3_ and SiO_2_ formed by the removal of hydroxyl groups can undergo a second hydration reaction with the cement hydration product Ca(OH)_2_, and this reaction, called the pozzolanic reaction, can generate C-S-H gel. During the pozzolanic reaction, Ca(OH)_2_ in the hydrated cement paste is consumed, and the amount of C-S-H gel increases. CANCP can also react with the hydration product C-S-H gel in the hydrated cement paste to create additional C-S-H called the pozzolanic C-S-H gel. The composition and properties of pozzolanic C-S-H gel and traditional C-S-H gel are different; the latter is relatively stable after polymerization with OH^-^ and Al^2+^ and can improve the performance of the traditional C-S-H gel to improve the performance of cement mortar, therefore improving the compressive strength of concrete. In Figure 8b, under a high-power electron microscope, C-S-H gel in concrete containing 10% CANCP is observed to grow dense and clustered to a higher degree than ordinary concrete. In addition, concrete containing 10% CANCP has a high degree of hydration.

The product of reactions between CANCP and concrete can improve the interface structure between cement paste and aggregate. A large amount of Ca(OH)_2_ exists in the interface area of concrete, which makes the interface a weak area. CANCP has a large specific surface area and a large surface energy and can rapidly react with Ca(OH)_2_ for secondary hydration reactions, reducing the content of Ca(OH)_2_ in the interface region, also decreasing the crystal size of Ca(OH)_2_ [44]. Therefore, the weak zone of the interface area is filled with hydration products and increases the strength. As shown in Figure 8 via high-power electron microscopy, a large amount of flake Ca(OH)_2_ is found in the hydrated cement paste of the ordinary concrete, but Ca(OH)_2_ is not found in the cement hydration paste of concrete containing 10% CANCP. CANCP can react with Ca(OH)_2_ for secondary hydration reactions and can consume the content of Ca(OH)_2_ in the interface region. 

The average particle size of CANCP is 23.35 μm, making it a relatively fine powder particle. Concrete is a granular accumulation system with continuous gradation, and the voids of each aggregate are filled with graded particles smaller than the voids. CANCP particles fill a wide range and can fill the fine pores in hardened cement paste, reducing the porosity of the paste. However, hydration reactions can produce hydrated calcium silicate and calcium sulphoaluminate hydrate with the filling effect and can optimize the internal pore structure of concrete, making the hardened cement paste and concrete stronger and denser. The hydration reactions can also strengthen the hardened cement paste and concrete to be more resistant to external deformation. Under low-magnification electron microscopy, the inner holes of the ordinary concrete are more numerous and are larger, and the surface morphology is not smooth or loose. Instead, there are few holes in the concrete containing 10% CANCP, and its surface is flat and dense.

## 4. Conclusions

The results of experimental studies on calcined underground ant nest materials were presented, and the following conclusions were drawn:

1. The results of chemical composition analysis indicate that the total contents of SiO_2_, Al_2_O_3_ and Fe_2_O_3_ for 800 °C CANCP and 600 °C CANCP reached 88.76% and 83.37%, respectively, which are both greater than the 70% specified in the ASTM standard.
2. It was shown via two methods (the lime absorption method and the strength index method) that CANCP has pozzolanic activity.
3. The strength contribution rate of the pozzolanic effect of mortar CANCP is positive and increases with age. CANCP has a favorable effect on the strength of the mortar system from an early age.
4. The compressive strength of concrete is maximized at 10% CANCP, and the early strength increases quickly. The compressive strength of concrete containing 20% CANCP is similar to that of the reference concrete without CANCP. When the content of CANCP is above 20%, the compressive strength of concrete begins to decrease, and the early strength decreases less.
5. The compressive strength of concrete with various CANCP content increases with age, and early strength develops faster. Increased strength is generally consistent with age when the CANCP content is not more than 15%.
6. The compressive strength of concrete containing CANCP with 800 °C calcination is higher than with calcination at 600 °C. CANCP with 800 °C calcination thus shows superior pozzolanic activity.
7. CANCP can improve the strength of mortar and concrete mainly because it can accelerate the hydration of cement and exhibits the pozzolanic and filling effects. Accelerating cement hydration and the pozzolanic effect is important for improving the strength of mortar and concrete, to which the filling effect also contributes in a secondary manner.

## Figures and Tables

**Table 1:** Chemical composition of materials (%) (calcined ant nest clay powder, CANCP).

Constituent	SiO_2_	Al_2_O_3_	Fe_2_O_3_	CaO	K_2_O	MgO	Na_2_O	SO_3_
Cement	23.25	7.79	2.41	46.78	0.574	0.459	0.574	4.10
Fly ash	47.14	22.67	1.02	4.28	0.721	6.25	0.468	2.3
Recycled powder	53.8	13.2	5.15	13.6	2.77	2.58	0.65	0.68
800 °C CANCP	69.5	14.1	5.16	2.27	2.81	2.24	1.34	0.14
600 °C CANCP	64.4	13.8	5.17	2.24	2.77	2.14	1.18	0.14

**Table 2:** Physical and mechanical properties of Portland cement.

Fineness (%)	Density (g/cm^3^)	Setting Time (min)		Flexural Strength (MPa)		Compressive Strength (MPa)	
		Initial Setting	Final Setting	3 Days	28 Days	3 Days	28 Days
5.7	3.13	150	490	5.5	7.6	24.6	44.3

**Table 3:** Physical and mechanical properties of fly ash (%).

Fineness	Water Requirement Ratio	Water Content	Loss on Ignition
18.8	92	0.2	6.9

**Table 4:** Physical and mechanical properties of recycled powder (%).

Loss on Ignition	Water Content	Soundness (mm)	Free Calcium Oxide	Water Requirement Ratio
5.9	0.5	0.5	0.35	105

**Table 5:** Proportion of mortar in each group (g).

Types of Mortar	Cement	Standard Sand	Water	Milled Standard Sand	Fly Ash	Recycled Powder	CANCP
CT	450	1350	225	-	-	-	-
SS	315	1485	225	-	-	-	-
GSS	315	1350	225	135	-	-	-
FA	315	1350	225	-	135	-	-
RP	315	1350	225	-	-	135	-
CAP	315	1350	225	-	-	-	135

**Table 6:** Flexural and compressive strength of mortar in each group (g).

Mortar Type	Flexural Strength (MPa)				Compressive Strength (MPa)				Strength Index (28 Days) (%)
	3 Days	7 Days	28 Days	90 Days	3 Days	7 Days	28 Days	90 Days	-
CT	5.23	6.40	7.73	9.73	28.18	38.85	51.92	62.15	100.0
SS	3.47	4.70	5.23	6.57	15.48	20.54	25.47	34.15	49.1
GSS	4.60	5.64	6.27	7.40	22.35	25.30	33.28	43.75	64.1
FA	3.93	5.27	7.20	9.47	18.88	28.20	37.33	53.68	71.9
RP	4.43	5.53	6.55	8.20	22.23	28.15	37.03	47.83	71.3
CAP	4.27	5.20	6.70	8.67	19.46	27.52	39.60	50.53	76.3

**Table 7:** Strength contribution rate of the pozzolanic effect.

Curing Time	Powder Type				
	SS	GSS	FA	RP	CAP
3 days	−27.42	11.73	−4.47	11.27	1.38
7 days	−32.40	−4.04	1.05	3.39	1.17
28 days	−42.70	−9.20	2.66	1.87	8.23
90 days	−27.39	0.56	18.96	11.93	13.91

**Table 8:** Basic material properties of the natural river sand.

Water Content (%)	Sediment Percentage (%)	Apparent Density (kg/m^3^)	Compact Packing Density (kg/m^3^)	Loose Packing Density (kg/m^3^)
2.0	1.5	2570	1760	1650

**Table 9:** Basic material properties of the natural stone.

Water Content (%)	Sediment Percentage (%)	Apparent Density (kg/m^3^)	Compact Packing Density (kg/m^3^)	Loose Packing Density (kg/m^3^)	Crushing Value (%)
0.8	0.9	2700	1430	1650	6.6

**Table 10:** Performance parameters of the water-reducing agents.

Solid Content (%)	Density	pH	Water-Reducing Rate of Concrete (%)	Fluidity of Cement Paste (mm)	Chloride Ion Content (%)	Total Alkalinity
40.19	1.090	6.0	30.0	285	0.05	1.2

**Table 11:** Mix proportion and slump (kg/m^3^).

Specimen Number	Cement	800 °C CANCP	600 °C CANCP	Sand	Stone	Water	Water Reducer	Slump
CT	486	0	0	741	1023	170	0.96	220
ZW_5_	461.7	24.3	0	741	1023	170	0.97	220
ZW_10_	437.4	48.6	0	741	1023	170	1.22	240
ZW_15_	413.1	72.9	0	741	1023	170	1.99	220
ZW_20_	388.8	97.2	0	741	1023	170	2.02	220
ZW_30_	340.2	145.8	0	741	1023	170	2.21	220
ZW600_10_	437.4	0	48.6	741	1023	170	1.01	230

**Table 12:** Relative compressive strength of CANCP concrete with age.

Specimen Name	Curing Days				
	3 Days	7 Days	14 Days	28 Days	90 Days
CT	0.604	0.711	0.846	1.000	1.104
ZW_5_	0.627	0.720	0.822	1.000	1.096
ZW_10_	0.619	0.696	0.852	1.000	1.087
ZW_15_	0.628	0.716	0.816	1.000	1.104
ZW_20_	0.625	0.724	0.831	1.000	1.052
ZW_30_	0.692	0.744	0.842	1.000	1.084
ZW600_10_	0.592	0.684	0.824	1.000	1.067
